# Editorial note: *CRABP2* promotes myoblast differentiation and is modulated by the transcription factors MyoD and Sp1 in C2C12 cells

**DOI:** 10.1371/journal.pone.0321709

**Published:** 2025-04-01

**Authors:** The PLOS One Editors

After this article [1] was published, concerns were raised that in Fig 1, the *CRABP1* and *CRBP2* panels appear similar, when levels are adjusted to visualize the background.

The corresponding author (KL) stated that the original image data underlying the published panels are unavailable; as such, this issue cannot be resolved. However, PLOS concluded that the article’s overall results and conclusions are not significantly affected, since the panels of concern present negative results and subsequent results relate to *CRABP2*.

The *PLOS One* Editors issue this Editorial Note to inform readers of this unresolved concern.
